# Taxifolin Attenuates Bacterial Pathogenicity and Thereby Enhances Antibacterial Efficacy in Clinically Relevant Bacteria

**DOI:** 10.3390/ijms27219469

**Published:** 2026-10-23

**Authors:** Ji-Hyun Yoon, Yeo-Jin Kim, Ki-Young Kim

**Affiliations:** 1 Graduate School of Biotechnology, Kyung Hee University, Seocheon, Giheung, Yongin 17104, Gyeonggi-do, Republic of Korea; hayop0909@khu.ac.kr; 2 Major in Bio-Cosmetic Engineering, Division of Convergence Studies, Sungkyul University, 53 Sungkyuldaehak-ro, Manan-gu, Anyang-si 14097, Gyeonggi-do, Republic of Korea

**Keywords:** taxifolin, biofilm-associated infections, antibiotic susceptibility, bacterial virulence, antimicrobial adjuvant

## Abstract

Biofilm formation is an important pathogenic process that contributes to bacterial virulence, persistence, and reduced susceptibility to antimicrobial treatment. Therefore, taxifolin was evaluated as a potential antibiofilm agent. Taxifolin was assessed for its effects on biofilm formation, planktonic growth, antibiotic susceptibility, and the expression of biofilm- and virulence-associated genes. Taxifolin inhibited biofilm formation by *Enterococcus faecalis*, *Enterococcus faecium*, *Escherichia coli*, *Streptococcus mutans*, *Streptococcus sobrinus*, *Staphylococcus aureus*, *Pseudomonas aeruginosa*, *Cutibacterium acnes*, and *Porphyromonas gingivalis* (IC50, 0.065~0.79 µg/mL). In *E. faecalis* and *E. faecium*, taxifolin did not substantially affect planktonic growth at 10 µg/mL. Taxifolin enhanced the antibacterial activity of commercially available antibiotics, and downregulated the expression of cytolysin-associated genes (*cylR*, *cylM*, and *cylLs*), quorum-sensing genes (*fsrB* and *fsrC*), biofilm-associated genes (*gelE*, *acm*, *scm*, *ebpA*, *ebpB* and *bps*), and the biofilm-associated virulence gene *Esp*. Taxifolin inhibited biofilm formation, enhanced bacterial susceptibility, and attenuated virulence-associated gene expression, supporting its potential as an adjunctive antibiofilm therapy for the management of biofilm-associated bacterial infections. Further studies are required to define its mechanism of action, activity against established biofilms, pharmacological properties, safety, and efficacy in appropriate in vivo models.

## 1. Introduction

**Figure 1:**
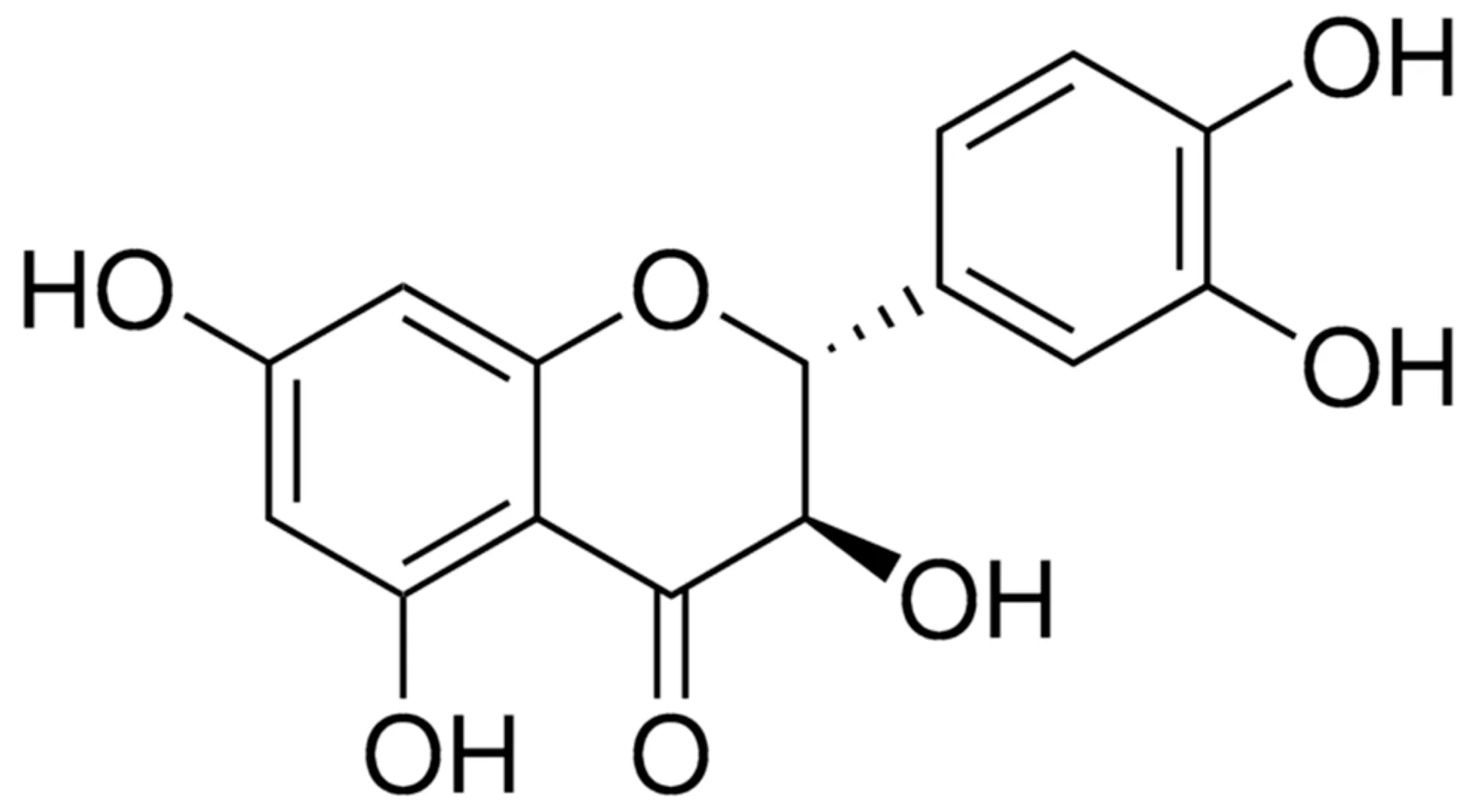
Chemical structure of taxifolin.

Bacterial biofilms are structured microbial communities in which bacterial cells are attached to biotic or abiotic surfaces and embedded within a self-produced extracellular polymeric substance (EPS) matrix. Biofilm development generally proceeds through several stages, including initial attachment, irreversible adhesion, maturation, and dispersion. During this process, bacteria undergo substantial physiological and transcriptional changes that promote persistence in hostile environments. The biofilm matrix provides mechanical stability and can restrict the penetration of antimicrobial agents, while nutrient and oxygen gradients generate metabolically heterogeneous bacterial populations with reduced susceptibility to antibiotics. Consequently, biofilm-associated infections are often difficult to eradicate and may persist despite conventional antimicrobial treatment [1,2,3,4,5]. Adhesion to host tissues is a critical first step that facilitates biofilm formation, which subsequently contributes to bacterial pathogenicity, colonization, immune evasion, and the establishment of chronic infections.Surface-associated bacterial cells are protected from host immune mechanisms and may tolerate antibiotic concentrations that are effective against planktonic cells. Moreover, bacteria within biofilms may exhibit reduced susceptibility to antimicrobial treatment, and biofilm-associated conditions may facilitate the exchange of genetic material associated with antimicrobial resistance. Therefore, inhibition of the early stages of biofilm formation represents an important strategy for preventing bacterial colonization and improving the efficacy of existing antibiotics [6,7,8,9,10]. Genes associated with biofilm formation may help predict the pathogenic potential of clinical enterococcal isolates. In *Enterococcus faecalis* and *Enterococcus faecium*, pili are encoded by a three-gene *ebp* locus that produces structural pilin subunits. Ebp pili mediate biofilm formation on abiotic surfaces and contribute to endocarditis and ascending urinary tract infections in experimental models. The enterococcal surface protein (*esp*) also promotes biofilm formation in both *E. faecalis* and *E. faecium*. Cytolysin is a pore-forming toxin produced by certain strains of *E. faecalis*.Multiple additional genes and gene clusters influence enterococcal biofilms, underscoring the complexity and multicomponent nature of this phenotype [11,12,13,14,15]. Natural flavonoids have attracted considerable attention as potential antibiofilm agents because they can interfere with bacterial adhesion, quorum sensing, virulence-associated pathways, EPS production, and cell-envelope integrity. Taxifolin, also known as dihydroquercetin, is a naturally occurring flavanonol found in several plant-derived materials (Figure 1). In addition to its antioxidant and anti-inflammatory properties, taxifolin has demonstrated antibacterial and antibiofilm activities against both Gram-positive and Gram-negative bacteria. However, the mechanisms underlying its antibiofilm activity may differ among bacterial species and remain incompletely characterized [16,17,18,19,20,21,22,23,24,25,26,27,28,29,30,31]. Despite their immense therapeutic potential, the clinical translation of flavonoids as anti-biofilm agents is often hindered by significant bottlenecks, including a limited understanding of their precise mechanisms of action, and a lack of comprehensive studies evaluating their combinatorial efficacy with conventional antibiotics across diverse panels of multidrug-resistant pathogens. Addressing these research gaps is crucial for the development of flavonoid-based adjunctive therapies. By elucidating the broad-spectrum antibiofilm activity of taxifolin and its synergistic potential, this study aims to provide foundational insights that could help future researchers design more effective, targeted strategies against biofilm-associated infections.

In the present study, we investigated the antibiofilm activity of taxifolin against a broad panel of bacterial species, including *E. faecalis*, *E. faecium*, *Escherichia coli*, *Streptococcus mutans*, *Streptococcus sobrinus*, *Staphylococcus aureus*, *Cutibacterium acnes*, *Porphyromonas gingivalis*, and *Pseudomonas aeruginosa*. We further examined whether taxifolin-mediated inhibition of biofilm formation influenced bacterial susceptibility to antibiotics. We hypothesized that taxifolin would suppress biofilm formation through mechanisms that impair bacterial attachment and biofilm development, thereby increasing antibiotic susceptibility. Our findings support the potential use of taxifolin as an antibacterial adjuvant for the prevention and treatment of biofilm-associated infections.

## 2. Results

### 2.1. Taxifolin Inhibits Biofilm Formation in Diverse Bacterial Species

**Figure 2:**
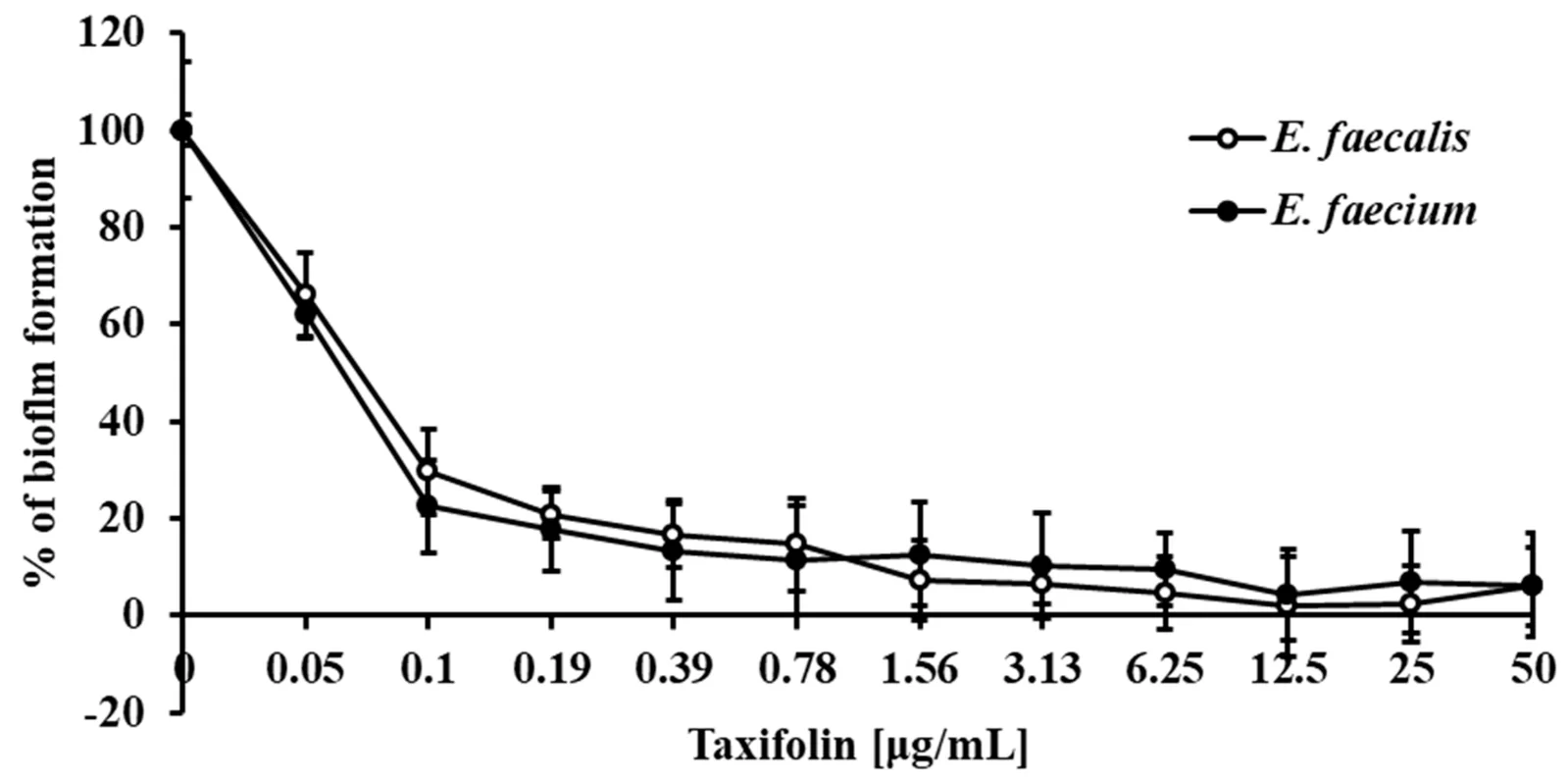
Taxifolin inhibits biofilm formation by enterococci. Biofilm formation of *E. faecalis* and *E. faecium* was induced in TSBg medium supplemented with the indicated concentrations of taxifolin and incubated at 37 °C for 24 h. Biofilm biomass was quantified by crystal violet staining (OD_595_) and expressed as a percentage of the untreated control. Data are presented as mean ± SD of at least three independent experiments.

Biofilm formation is a critical determinant of bacterial survival, persistence, and pathogenicity. To evaluate the antibiofilm activity of taxifolin, we assessed its effects on biofilm formation across nine bacterial species representing both Gram-positive and Gram-negative pathogens. Taxifolin exhibited dose-dependent inhibition of biofilm formation in all tested species, with half-maximal inhibitory concentrations (IC_50_) ranging from 0.065 to 0.79 μg/mL (Figure 2 and Figure S1, and Table 1).

Notably, taxifolin demonstrated potent activity against enterococcal species, with IC_50_ values of 0.065 μg/mL for *E. faecium* and 0.081 μg/mL for *E. faecalis*. In comparison, IC_50_ values for other species were as follows: *S. aureus* (0.099 μg/mL), *S. mutans* (0.11 μg/mL), *S. sobrinus* (0.18 μg/mL), *P. aeruginosa* (0.34 μg/mL), *P. gingivalis* (0.41 μg/mL), *E. coli* (0.63 μg/mL), and *C. acnes* (0.79 μg/mL) (Figure 2 and Figure S1 and Table 1).

Given the superior efficacy of taxifolin against *E. faecalis* and *E. faecium*, combined with the clinical significance of enterococci as major pathogens associated with nosocomial infections, the vancomycin-resistant *E. faecalis* strain (CCARM 5511) and the standard reference *E. faecium* strain (KACC 11954) were selected for subsequent mechanistic investigations.

### 2.2. Taxifolin Enhances Antibiotic Susceptibility in E. faecium and E. faecalis

Antibiotics remain the primary therapeutic approach for controlling *E. faecalis* and *E. faecium* infections; however, biofilm formation is a well-established contributor to antibiotic tolerance and treatment failure. To investigate whether taxifolin could potentiate the activity of conventional antibiotics, we evaluated bacterial viability in biofilms treated with ampicillin (AMP), vancomycin (VAN), or oxytetracycline (OXY) in the presence or absence of taxifolin (TF) for 24 h.

**Figure 3:**
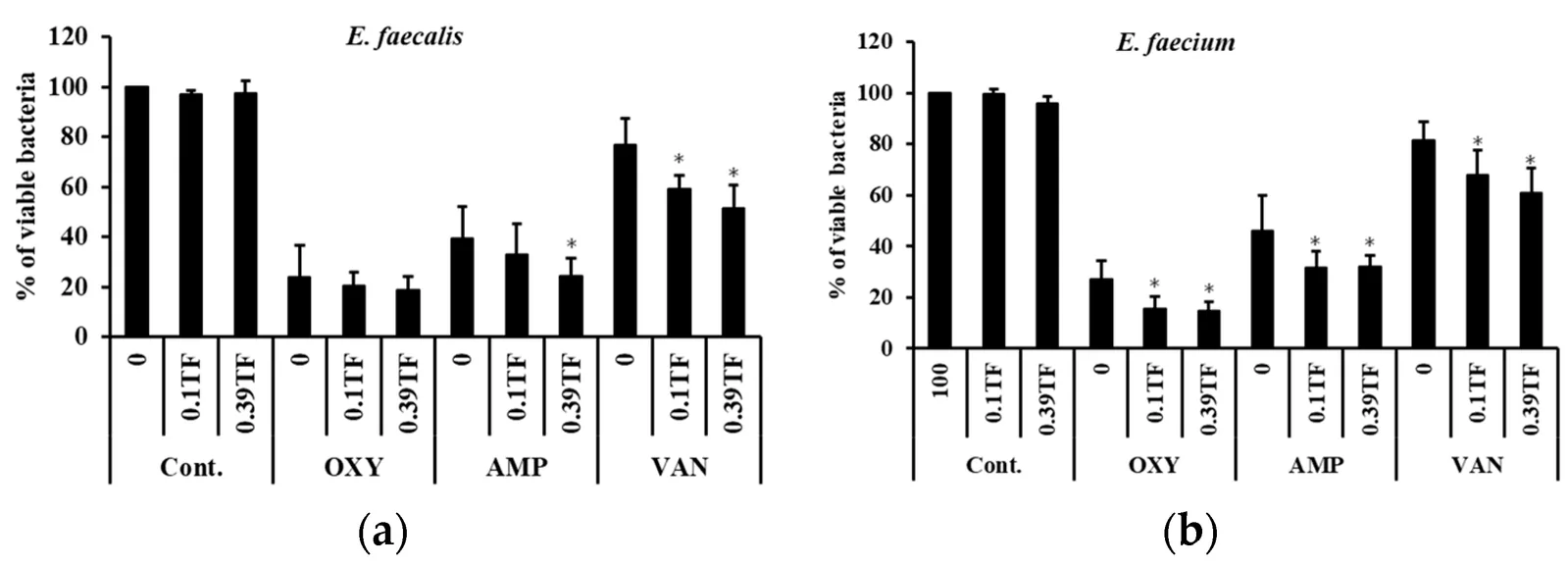
Taxifolin enhances antibiotic susceptibility in enterococcal biofilms. (**a**) The antibacterial activity of oxytetracycline (OXY), ampicillin (AMP), and vancomycin (VAN) against *E. faecalis* biofilms was evaluated in the presence or absence of taxifolin. *E. faecalis* biofilms were established in TSBg for 24 h and subsequently treated with OXY (3.125 μg/mL), AMP (6.25 μg/mL), and VAN (100 μg/mL), either alone or in combination with taxifolin at 0.1 (0.1 TF) or 0.39 (0.39 TF) μg/mL, for an additional 24 h. (**b**) The same treatment strategy was used to assess the effect of taxifolin on antibiotic activity against *E. faecium* biofilms. *E. faecium* biofilms were formed in TSBg for 24 h and then incubated with OXY (3.125 μg/mL), AMP (6.25 μg/mL), and VAN (100 μg/mL), either alone or in combination with taxifolin at 0.1 (0.1 TF) or 0.39 (0.39 TF) μg/mL, for 24 h. Statistical significance was determined using the indicated statistical analysis (* *p* < 0.05, *p* < 0.001).

Taxifolin significantly enhanced the antibacterial activity of all tested antibiotics against both the vancomycin-resistant *E. faecalis* (CCARM 5511) and the standard reference *E. faecium* (KACC 11954) strains (Figure 3). Although antibiotic monotherapy reduced viable-cell counts, culturable bacteria remained detectable after 24 h of treatment (Figure 3).

In *E. faecalis*, treatment with 0.39 μg/mL taxifolin in combination with ampicillin reduced viable cell counts (assessed by CFU enumeration) to 24.59%, compared with 39.6% viability with ampicillin alone. Similarly, the combination of taxifolin with oxytetracycline reduced viability to 19.0%, compared with 24.0% with oxytetracycline monotherapy. Notably, even against vancomycin-resistant *E. faecalis*, the addition of 0.39 μg/mL taxifolin to vancomycin reduced bacterial viability by 51.6%, demonstrating the capacity of taxifolin to overcome antibiotic resistance (Figure 3a).

In the standard reference strain *E. faecium* (KACC 11954), treatment with 0.39 μg/mL taxifolin significantly potentiated the activity of ampicillin, oxytetracycline, and vancomycin, resulting in marked reductions in biofilm viability (as determined by CFU enumeration) compared with antibiotic treatment alone (Figure 3b). These findings indicate that taxifolin can restore or enhance antibiotic efficacy against enterococcal biofilms in both an antibiotic-resistant strain (*E. faecalis* CCARM 5511) and a standard reference strain (*E. faecium* KACC 11954).

### 2.3. Taxifolin Does Not Affect Bacterial Growth

Certain antibacterial compounds inhibit biofilm formation indirectly by exerting bactericidal or bacteriostatic effects, thereby reducing the bacterial population available for biofilm development. To determine whether the observed inhibition of biofilm formation by taxifolin was attributable to direct antibacterial activity, we evaluated the effects of taxifolin on bacterial growth over 72 h using a high concentration of 10 μg/mL. This concentration was deliberately selected as a stringent test condition because it is more than 100-fold higher than the biofilm inhibitory IC50 values (0.065–0.081 μg/mL) and well above the working concentrations used in the combinatorial assays (0.39 μg/mL).

**Figure 4:**
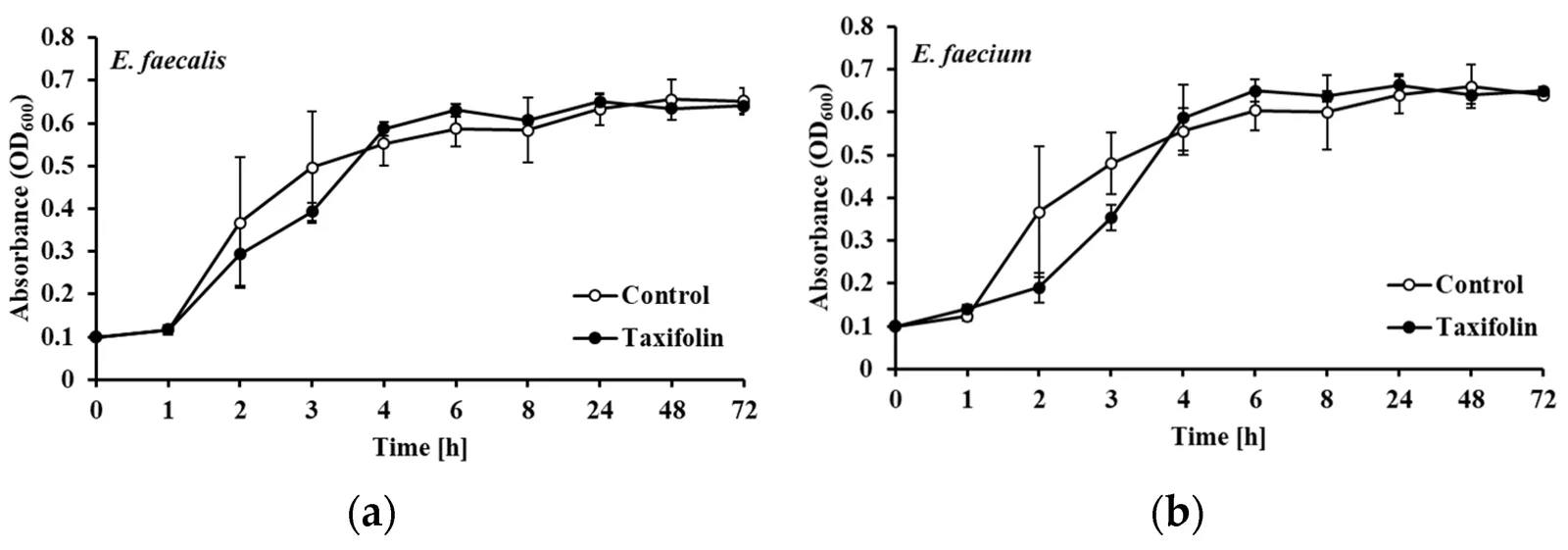
Taxifolin did not inhibit the growth of *E. faecalis* or *E. faecium*. Cultures of *E. faecalis* (**a**) and *E. faecium* (**b**), each adjusted to an initial OD_600_ of 0.1, were incubated with taxifolin at 10 μg/mL for 72 h at 37 °C.

Taxifolin, even at 10 μg/mL, did not significantly affect the growth of *E. faecalis* or *E. faecium* compared with untreated controls (Figure 4). These results indicate that the antibiofilm activity of taxifolin is not secondary to growth inhibition or bactericidal effects, but rather reflects a specific interference with biofilm formation processes.

### 2.4. Taxifolin Suppresses Expression of Biofilm-Associated and Virulence Genes

To investigate the molecular basis of taxifolin-mediated inhibition of biofilm formation, we used quantitative real-time PCR (qRT-PCR) to assess the expression of genes involved in biofilm development, quorum sensing (QS), and virulence in *E*. *faecalis* and *E*. *faecium*.

**Figure 5:**
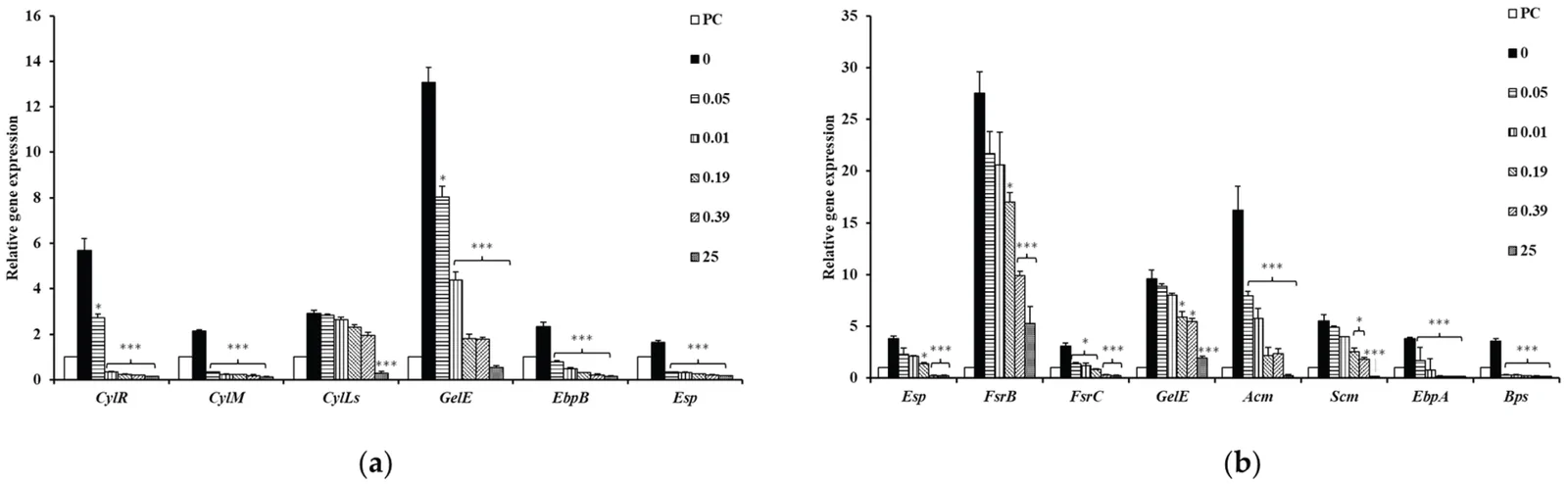
Taxifolin suppressed the expression of genes associated with biofilm formation and virulence in *E. faecalis* and *E. faecium*. (**a**) Taxifolin dose-dependently suppressed the expression of genes involved in enterococcal cytolysin biosynthesis, and quorum-sensing and biofilm-formation, including *cylR*, *cylM*, *cylLs*, *gelE*, *ebpB* and *esp*, in *E. faecalis*. (**b**) In *E. faecium*, taxifolin decreased the expression of genes involved in quorum-sensing and biofilm-formation, including *esp*, *fsrB*, *fsrC*, *gelE*, *acm*, *scm*, *ebpA*, and *bps*. PC represents planktonic cells, whereas “0” represents the untreated biofilm control. Number indicates the treated concentration of taxifolin (μg/mL). Data are presented as mean ± SD. Statistical significance was determined using the indicated statistical analysis (* *p* < 0.05, *** *p* < 0.001).

Taxifolin treatment produced a dose-dependent decrease in the transcription of several biofilm-associated genes. Specifically, genes encoding adhesins and biofilm matrix-related components in *E. faecalis*, including *gelE*, *ebpB*, and *esp*, were significantly downregulated following taxifolin exposure (Figure 5). Taxifolin also suppressed the expression of *esp*, *gelE*, *acm*, *scm*, *ebpA*, *bps, fsrB* and *fsrC* in *E. faecium*, suggesting that the compound interferes with cell-to-cell signaling pathways involved in biofilm regulation (Figure 5). Furthermore, taxifolin markedly reduced the expression of genes associated with enterococcal cytolysin production (*cylR*, *cylM*, and *cylLs*) in *E. faecalis*, indicating substantial suppression of this important virulence determinant. Overall, these results suggest that taxifolin inhibits biofilm formation by transcriptionally repressing genes involved in biofilm matrix production, quorum-sensing regulation, and virulence-factor expression.

## 3. Discussion

Biofilm-associated infections caused by both Gram-positive and Gram-negative bacteria remain a major therapeutic challenge. Clinically important organisms, including *E. faecalis*, *E. faecium*, *E. coli*, *P. gingivalis*, *S. mutans*, *S. sobrinus*, *S. aureus*, *C. acnes*, and *P. aeruginosa*, can establish structured microbial communities that promote persistence and reduce susceptibility to antimicrobial therapy. Biofilm-associated tolerance is multifactorial and may involve restricted antimicrobial penetration, altered metabolic activity, stress-response pathways, phenotypic heterogeneity, and protection within the extracellular polymeric substance matrix. In enterococci, this phenotype is particularly important because *E. faecalis* and *E. faecium* are associated with healthcare-associated infections and may carry multiple antimicrobial-resistance determinants, including determinants affecting glycopeptide and β-lactam susceptibility. However, biofilm formation and antimicrobial resistance are related but distinct phenotypes; therefore, biofilm inhibition should be considered a complementary strategy rather than a substitute for conventional antimicrobial susceptibility testing or resistance surveillance [1,2,3,4,5,6,7,8,9,10].

Natural products are increasingly being investigated as sources of anti-biofilm molecules because they may interfere with bacterial adhesion, signaling, matrix production, and virulence without necessarily exerting strong bactericidal pressure [9,10,32,33,34]. Furthermore, the lack of planktonic growth inhibition is a strategic advantage of anti-virulence agents like taxifolin. By disarming the biofilm defense without affecting bacterial viability, taxifolin minimizes the selective pressure that typically drives antimicrobial resistance, reinforcing its clinical suitability as an ideal adjunctive therapy. Flavonoids are particularly attractive in this context because of their structural diversity and reported antioxidant, anti-inflammatory, antibacterial, antiviral, and anticancer activities [35]. Taxifolin, also known as dihydroquercetin, is a flavanonol that has been associated with several pharmacological effects. Importantly, taxifolin is a widely distributed natural compound with a well-documented safety profile [16,17]. Previous toxicological and pharmacological studies have demonstrated that taxifolin exhibits favorable safety profiles regarding cytotoxicity in normal human cells [18], genotoxicity and mutagenicity [19,20], hypoallergenicity [21,22], and immunotoxicity [23]. However, the exposure dose resulting from oral administration as a dietary supplement differs substantially from the concentrations required for direct contact with an infected site or for systemic therapy. Although recent studies have supported the biocompatibility and safety of taxifolin in topical skin and direct wound-contact applications [22,24] as well as in systemic administration against experimental bacterial infections in vivo [30], route- and dose-specific toxicological evaluations remain essential for clinical translation. Furthermore, the biological activity of taxifolin is strongly dependent on the bacterial species, strain, concentration, exposure time, and experimental model. Consequently, its activity should be interpreted in relation to the tested bacterial population and the distinction between inhibition of planktonic growth, prevention of biofilm formation, and disruption of pre-existing biofilms [25,26,27,28,29,30,31].

In the present study, taxifolin showed the inhibitory activity of biofilm formation by all tested bacterial species, including *E. faecalis*, *E. faecium*, *E. coli*, *P. gingivalis*, *S. mutans*, *S. sobrinus*, *S. aureus*, *C. acnes*, and *P. aeruginosa* (Figure 2 and Figure S1 and Table 1). This broad activity is notable because the panel included organisms with distinct cell-envelope structures, ecological niches, and biofilm-associated regulatory systems. The findings suggest that taxifolin may act on conserved processes required for biofilm development, such as initial surface attachment, cell aggregation, extracellular-matrix production, or intercellular signaling. However, the apparent broad-spectrum activity should not be interpreted as evidence of a single universal molecular target. The mechanisms controlling biofilm development differ substantially among the tested species, and species-specific validation will be required to determine whether taxifolin acts through a common pathway or through different targets in different bacteria.

The antibiotic-combination experiments provide additional evidence that taxifolin may improve antimicrobial activity under biofilm-forming conditions. Vancomycin alone did not substantially reduce the growth of *E. faecalis* or *E. faecium* within biofilms, whereas the combination of vancomycin and taxifolin reduced bacterial growth by approximately 51.6 and 60.9% of *E. faecalis* and *E. faecium, respectively*. This observation is consistent with the concept that disruption of the biofilm state can increase bacterial exposure to antibiotics or render cells metabolically more susceptible to antibiotic-mediated killing. However, the result should be described as an enhancement of vancomycin activity rather than definitive synergy unless synergy was formally evaluated. A quantitative interaction analysis, such as a checkerboard assay, a time–kill analysis, or a biofilm-specific combination model, would be required to distinguish synergy from additivity or independent effects.

Importantly, CFU enumeration after antibiotic treatment does not establish antibiotic persistence or identify the mechanisms underlying the survival of the remaining bacteria. Therefore, the viable bacteria recovered in this study are described as residual viable bacteria rather than persister cells. Further studies using time-kill assays and assessments of regrowth and antibiotic susceptibility would be required to specifically investigate antibiotic persistence.

The difference between prevention of biofilm formation and eradication of an established biofilm is also important. Biofilm formation assays primarily evaluate early-stage attachment and development, whereas mature biofilms contain an established extracellular matrix and physiologically heterogeneous bacterial populations that may exhibit reduced susceptibility to antibiotics. An agent that effectively prevents biofilm formation may therefore have limited activity against a mature biofilm. This distinction is particularly relevant to the present study because taxifolin has been reported to reduce *S. aureus* biofilm formation through inhibition of sortase A [30], while its activity against mature biofilms may be more limited. However, it is crucial to note that while taxifolin monotherapy may have limited eradication efficacy against established biofilms, our combinatorial assays (Figure 3) demonstrated that taxifolin successfully sensitized pre-formed, mature biofilms to conventional antibiotics. This suggests that its primary clinical value lies in its synergistic role as an adjunctive agent to compromise mature biofilm defenses, rather than as a standalone eradicating agent.

The observed effects on biofilm-associated gene expression further suggest that taxifolin influences bacterial regulatory processes. In this study, taxifolin reduced the expression of genes related to quorum sensing, adhesion, colonization, virulence, and antimicrobial resistance (Figure 5). These changes are compatible with a model in which taxifolin suppresses the transcriptional programs required for the transition from planktonic growth to a surface-associated community. Quorum-sensing pathways are particularly relevant because they coordinate population-dependent behaviors, including motility, extracellular-matrix production, virulence-factor expression, and biofilm maturation. Nevertheless, reduced transcript abundance does not establish that a gene product is directly inhibited by taxifolin. Because taxifolin did not inhibit general planktonic growth (Figure 4), a universal bactericidal mechanism—such as the depletion or direct global inhibition of essential RNA polymerases—is highly unlikely. Instead, we hypothesize that taxifolin may act as an antagonist to specific quorum-sensing sensor kinases (e.g., the Fsr system in enterococci) or directly inhibit critical surface-anchoring enzymes (such as sortase A) that regulate biofilm matrix production. The observed downregulation of biofilm-associated genes is likely a downstream transcriptional consequence of this primary signal interference. Elucidating the precise direct molecular binding targets of taxifolin—using advanced techniques such as molecular docking, surface plasmon resonance (SPR), and targeted proteomics—represents a critical direction for our future mechanistic studies.

The relationship between biofilm formation, virulence, and antimicrobial resistance also requires careful interpretation. A reduction in the expression of resistance-associated genes does not necessarily mean that taxifolin reverses the corresponding resistance phenotype. Conversely, increased antibiotic susceptibility in a taxifolin-treated biofilm may result from matrix disruption, altered metabolic activity, increased antibiotic penetration, or reduced cell density without any change in the underlying resistance determinant. Future experiments should therefore combine transcriptional analysis with phenotypic measurements, including MIC, minimum biofilm inhibitory concentration, minimum biofilm eradication concentration, viable-cell enumeration, matrix quantification, antibiotic penetration, and expression or activity of the relevant resistance proteins.

Overall, the present findings identify taxifolin as a promising inhibitor of biofilm development across a diverse group of pathogenic bacteria and show that it can enhance antibiotic susceptibility against *E. faecalis* and *E. faecium* biofilms. The results support further investigation of taxifolin as an adjunctive anti-biofilm agent, particularly in combination with conventional antibiotics. However, the current evidence does not yet establish a universal mechanism of action or demonstrate efficacy against established biofilm-associated infections in vivo. A limitation of the present study is that only a single strain of each bacterial species was evaluated. Given the genetic and phenotypic diversity among clinical isolates, future studies incorporating multiple clinical strains and diverse isolates are necessary to confirm the broad applicability and strain-independent efficacy of taxifolin. Future studies should define the concentration–response relationship, determine whether taxifolin is active against mature biofilms, formally quantify antibiotic interactions, validate species-specific molecular targets, assess cytotoxicity and pharmacokinetics, and evaluate efficacy in appropriate animal models. Such studies will be necessary to determine whether taxifolin can progress from an in vitro biofilm inhibitor to a clinically relevant adjunct for treatment of persistent bacterial infections. Additionally, future investigations should incorporate high-resolution microscopic analyses, such as field emission scanning electron microscopy (FESEM), atomic force microscopy (AFM), and Live/Dead fluorescence imaging, to visually evaluate and confirm the structural and morphological disruptions induced by taxifolin within the biofilm architecture.

## 4. Materials and Methods

### 4.1. Bacterial Strains and Culture Conditions

All experimental procedures and materials used in this study were performed as previously described in our earlier publication [9,10].

The bacterial strains used in this study are listed in Table 1. Notably, the *Enterococcus faecalis* (CCARM 5511) strain is a vancomycin-resistant enterococcus (VRE) isolate, whereas the *Enterococcus faecium* (KACC 11954) strain is a standard reference strain and not a multidrug-resistant (MDR) strain. *E. faecalis*, *E. faecium*, *E. coli*, and *S. aureus* were maintained in tryptic soy broth supplemented with 1% glucose (TSBg). *S*. *mutans* and *S*. *sobrinus* were cultured in brain heart infusion (BHI) broth. *P*. *gingivalis* was maintained in tryptic soy broth supplemented with 10% defibrinated horse blood agar under anaerobic conditions. *P*. *aeruginosa* was maintained in nutrient agar. *C*. *acnes* was cultured in reinforced clostridial agar and incubated at 37 °C under anaerobic conditions [9,10,36,37,38].

### 4.2. Biofilm Formation Assay

Biofilm formation assays were performed in the medium used to maintain the bacteria. Bacterial suspensions were adjusted to an optical density at 600 nm (OD_600_) of 0.1 and inoculated into 96-well flat-bottomed microtiter plates. Taxifolin was added at the indicated concentrations, and plates were incubated for 24 h at 37 °C. Following incubation, wells were washed three times with 200 μL of phosphate-buffered saline (PBS). Biofilms were stained with 100 μL of 1% aqueous crystal violet solution for 30 min at room temperature, washed three times with 200 μL of PBS, and destained with 150 μL of 30% acetic acid for 15 min. The absorbance of the destained solution was measured at 595 nm using a microplate reader (BioTek Instruments, Winooski, VT, USA) [9,10,36,37,38].

### 4.3. Evaluation of Combinatorial Antibacterial Activity

The antibacterial effects of combinatorial treatment with taxifolin and conventional antibiotics were evaluated using the plate counting method. Biofilms of *E. faecium* and *E. faecalis* were prepared as described above. After biofilm formation, the culture medium was gently removed, and the pre-formed biofilms attached to the surface were incubated with 1 mL of fresh medium containing ampicillin (6.25 μg/mL), vancomycin (100 μg/mL), or oxytetracycline (3.125 μg/mL) in the presence of indicated concentrations of taxifolin for 24 h. Control groups received antibiotics alone without taxifolin. Following treatment, biofilm cells were harvested, serially diluted in phosphate-buffered saline, and plated on LB agar. Colony-forming units (CFUs) were enumerated after incubation at 37 °C for 24 h [9,10,37].

### 4.4. Bacterial Growth Curve Analysis

Bacterial growth curves were generated as previously described with minor modifications [9,10]. *E. faecalis* and *E. faecium* cultures were prepared in tryptic soy broth (TSB) and adjusted to an optical density at 600 nm (OD_600_) of 0.1. Taxifolin (10 μg/mL) was added to the cultures, which were then incubated at 37 °C. Bacterial growth was monitored by measuring the optical density at 600 nm at the indicated time intervals using a microplate reader (BioTek Instruments, Winooski, VT, USA).

### 4.5. Quantitative Real-Time PCR (qRT-PCR) Analysis

Bacterial cultures and taxifolin treatment conditions were identical to those described for the biofilm formation inhibition assay. Total RNA was isolated using TRIzol reagent (Life Technologies, Thermo Fisher Scientific, Waltham, MA, USA) according to the manufacturer’s instructions. Reverse transcription was performed using 1 μg of total RNA with a reverse transcriptase kit (NanoHelix, Daejeon, Republic of Korea) to synthesize complementary DNA (cDNA). Quantitative real-time PCR (qRT-PCR) was carried out using 2× SybrGreen qPCR Master Mix (CellSafe, Seoul, Republic of Korea). Primer sequences are listed in Table 2. The genes encoding 23S rRNA and *tufA* were used as endogenous controls for normalization. Relative gene expression levels were calculated using the 2^−ΔΔCt^ method.

### 4.6. Statistical Analysis

All experiments were done independently at least three times. Data are presented as mean ± standard deviation (SD). The normal distribution of the data was verified using the Shapiro–Wilk test prior to statistical analysis. Comparisons between groups were performed using one-way ANOVA followed by Student’s *t*-test. A *p*-value of <0.05 was considered statistically significant. Statistical annotations are denoted as * *p* < 0.05, ** *p* < 0.01, and *** *p* < 0.001.

## 5. Conclusions

In conclusion, taxifolin significantly inhibited biofilm formation by a broad range of clinically relevant bacterial pathogens. In *E. faecalis* and *E. faecium*, planktonic growth was not substantially affected at 10 μg/mL. Taxifolin also enhanced the antibacterial activity of conventional antibiotics and downregulated genes associated with quorum sensing, adhesion, biofilm development, cytolysin production, and bacterial virulence. These findings indicate that taxifolin may interfere with biofilm-associated pathogenicity and could serve as a promising adjunctive anti-biofilm agent to improve the treatment of persistent bacterial infections. However, further studies are required to clarify its species-specific mechanisms of action, evaluate its activity against established biofilms, determine its safety and pharmacokinetic properties, and confirm its therapeutic efficacy in appropriate in vivo models.

## Figures and Tables

**Table 1:** Bacterial strains used in this study and IC_50_ values of taxifolin for inhibition of biofilm formation.

Strain	Strain Number	IC_50_ [μg/mL]	Source
*Enterococcus faecalis* (Vancomycin-resistant)	CCARM5511	0.081	Purchased from KACC (Korean Agricultural Culture Collection), CCARM (Culture Collection of Antimicrobial Resistant Microbes), or KCTC (Korean Collection for Type Cultures)
*Enterococcus faecium*	KACC11954	0.065	
*Escheri* *c* *hia coli*	KACC11598	0.63	
*Streptococcus mutans*	KACC16833	0.11	
*Streptococcus sobrinus*	CCARM3506	0.18	
*Staphylococcus aureus*	KCTC5809	0.099	
*Pseudomonas aeruginosa*	KACC14021	0.34	
*Cutibacterium acnes*	CCARM9009	0.79	
*Porphyromonas gingivalis*	KCTC5352	0.41	

**Table 2:** Primers used in this study.

Genes	Primer Sequence: 5′ to 3′	Function	Reference
For *E. faecalis*			
*cylR*	F: TTTATTTTTATTGGATATCATTCTGTAGTC R: TTCGCTCATCTTTTTTGAATACAG	Cytolysin regulatory	[36]
*cylM*	F: TCGGACACGGTATATATAGCTATGT R: TCTACTAGTGTACTTTGATTACCATAATAATT	Cytolysin toxin	[36]
*cylLs*	F: CTGTTGCGGCGACAGCT R: CCACCAACCCAGCCACAA	Cytolysin toxin	[36]
*gelE*	F: CGGAACATACTCAACGTTTGA R: TGGATTAGATGCACCCGAAAT	Gelatinase	[36]
*ebpB*	F: CGTACAGGAGGCAAGTCTTT R: AGGTATTCCCCGCTTGATTT	Biofilm-associated pili	[36]
*esp*	F: GCATCAGTATTAGTTGGT R: TTCCTTGTAACACATCAC	Biofilm formation	[36]
For *E. faecium*			
*esp*	F: CCACGAGTTAGAGGGAACAG R: TTGGAGCCCCATCTTTTTCA	Biofilm formation	[39]
*fsrB*	F: TGCTCAAAAAGCAAAGCCTTATAA R: GATGACGAGACCGTAGAGTATTACTGAA	Efae regulator	[40]
*fsrC*	F: GCTTATTTGGAAGAACAACGTATCAA R: CGAAACATCGCTAGCTCTTCGT	Efae regulator	[40]
*gelE*	F: CGGAACATACTGCCGGTTTAGA R: TGGATTAGATGCCACCCGAAAT	Gelatinase	[40]
*acm*	F: TCAGCAGTAATGTCACTTCGTTG R: GAATAGGCTGTTCATCTGCTCG	Collagen-binding adhesin	[41]
*scm*	F: CTAACTGGTAACTATGGCTTGT R: GTCCGTGCTGTCACTTGT	Collagen-binding adhesin	[41]
*ebpA*	F: ACCAAGCCAGACGAAATAGAAGAAG R: ATTGTTTTGGTCAGGTGCATCATAGA	Biofilm-associated pili	[11]
*bps*	F: TATCAGCAACAAGCGGTCAA R: AATCCTGCCCTTTTTCGATT	Biofilm formation	[11]
*tufA*	F: TACACGCCACTACGCTCAC R: AGCTCCGTCCATTTGAGCAG	Housekeeping gene	[39]
23S rRNA	F: CCTATCGGCCTCGGCTTAG R: AGCGAAAGACAGGTGAGAATCC	Housekeeping gene	[36]

## Data Availability

Data are contained within the article.

## Supplementary Materials

The following supporting information can be downloaded at: https://www.mdpi.com/article/10.3390/ijms27219469/s1.

## Abbreviations

The following abbreviations are used in this manuscript:

IC_50_	Half-Maximal Inhibitory Concentration
KACC	Korean Agricultural Culture Collection
CCARM	Culture Collection of Antimicrobial Resistant Microbes
KCTC	Korean Collection for Type Cultures
TSB	Tryptic soy broth
LB	Luria–Bertani Medium
BHI	Brain Heart Infusion Medium
AMP	Ampicillin
VAN	Vancomycin
OXY	Oxytetracycline
TF	Taxifolin
PBS	Phosphate-buffered saline
CFU	Colony-forming unit
