# Supplementary material for: Taxifolin Attenuates Bacterial Pathogenicity and Thereby Enhances Antibacterial Efficacy in Clinically Relevant Bacteria
Source: Int J Mol Sci. 2026 Oct 23;27(21):9469. doi: 10.3390/ijms27219469 (PMC13762615; doi:10.3390/ijms27219469)
Supplement: Supplementary file 1 [file ijms-27-09469-s001.zip › ijms-4596677-supplementary.pdf]

Fig. S1a. Taxifolin inhibits biofilm formation by *Staphylococcus aureus*

Fig. S1b. Taxifolin inhibits biofilm formation by *Streptococcus mutans*

Fig. S1c. Taxifolin inhibits biofilm formation by *Pseudomonas aeruginosa*

Fig. S1d. Taxifolin inhibits biofilm formation by *Escherihia coli*

Fig. S1e. Taxifolin inhibits biofilm formation by *Cutibacterium acnes*

Fig. S1f. Taxifolin inhibits biofilm formation by *Porphyromonas gingivalis*

Fig. S1g. Taxifolin inhibits biofilm formation by *Streptococcus sobrinus*

Fig. S1.

(A)

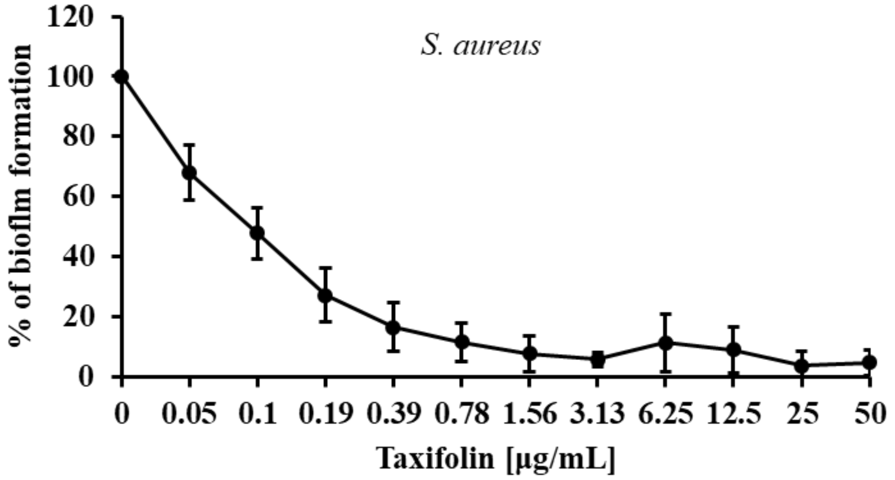

S. aureus
% of bioflm formation
120
100
80
60
40
20
0
0.05
0.1
0.19
0.39
0.78
1.56
3.13
6.25
12.5
25
50
Taxifolin [μg/mL]

(B)

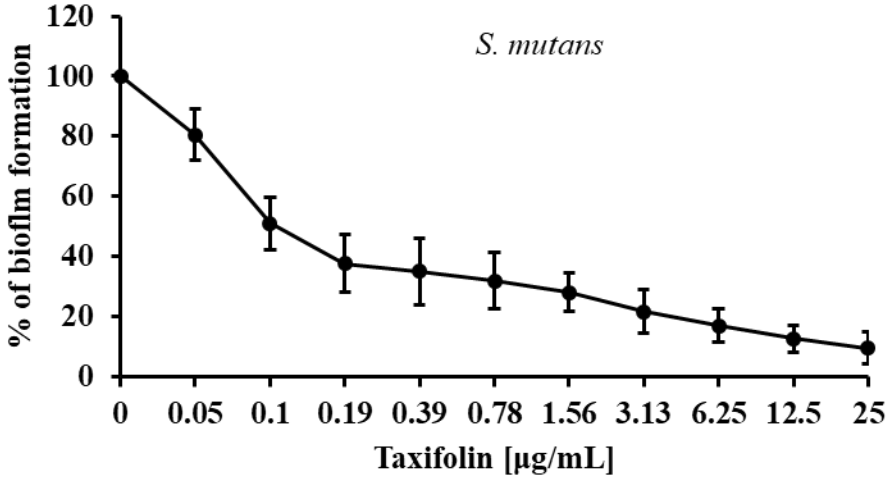

S. mutans
% of bioflm formation
120
100
80
60
40
20
0
0.05
0.1
0.19
0.39
0.78
1.56
3.13
6.25
12.5
25
Taxifolin [μg/mL]

(C)

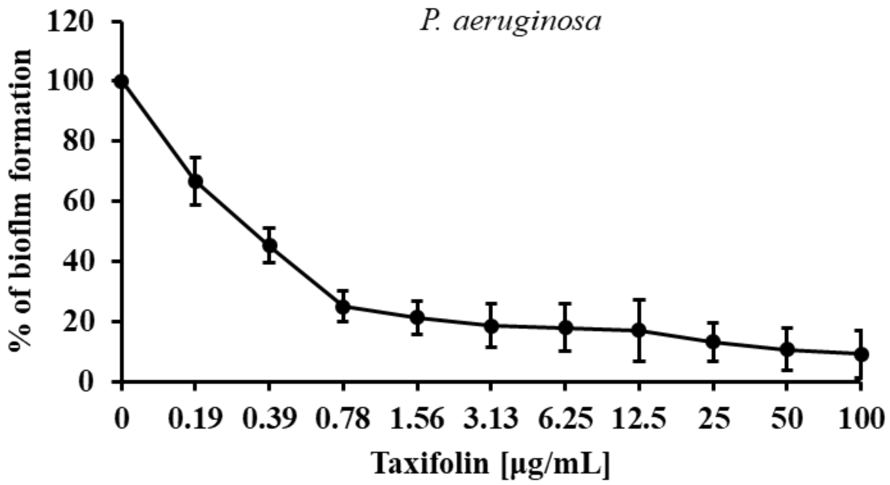
P. aeruginosa
120
100
80
60
40
20
0
% of bioflm formation
0
0.19
0.39
0.78
1.56
3.13
6.25
12.5
25
50
100
Taxifolin [μg/mL]

(D)

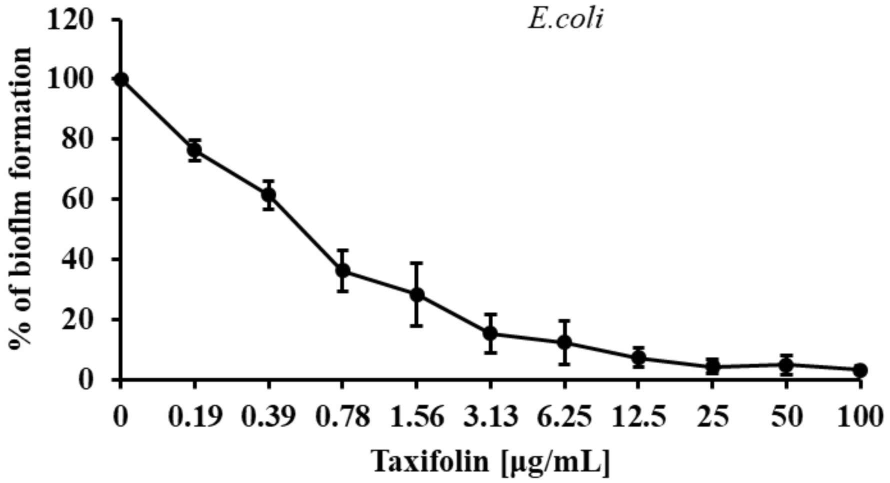
E.coli
120
100
80
60
40
20
0
% of bioflm formation
0
0.19
0.39
0.78
1.56
3.13
6.25
12.5
25
50
100
Taxifolin [μg/mL]

(E)

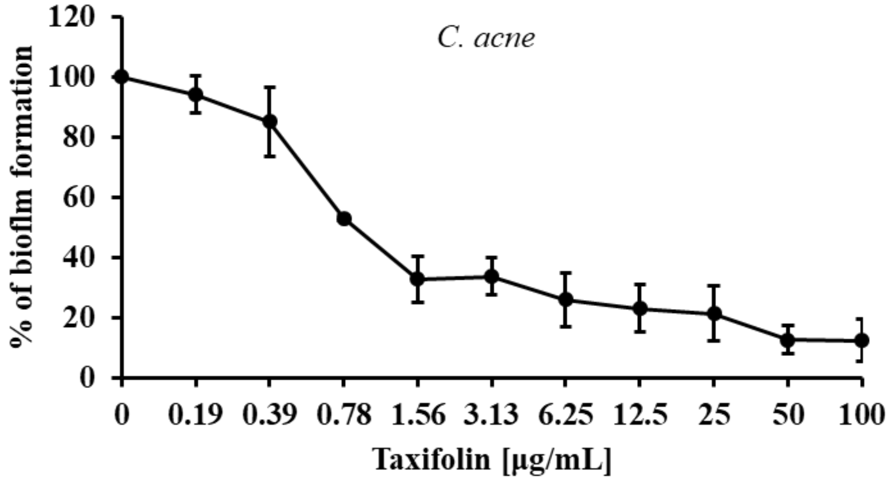
C. acne
120
100
80
60
40
20
0
% of bioflm formation
0
0.19
0.39
0.78
1.56
3.13
6.25
12.5
25
50
100
Taxifolin [µg/mL]

(F)

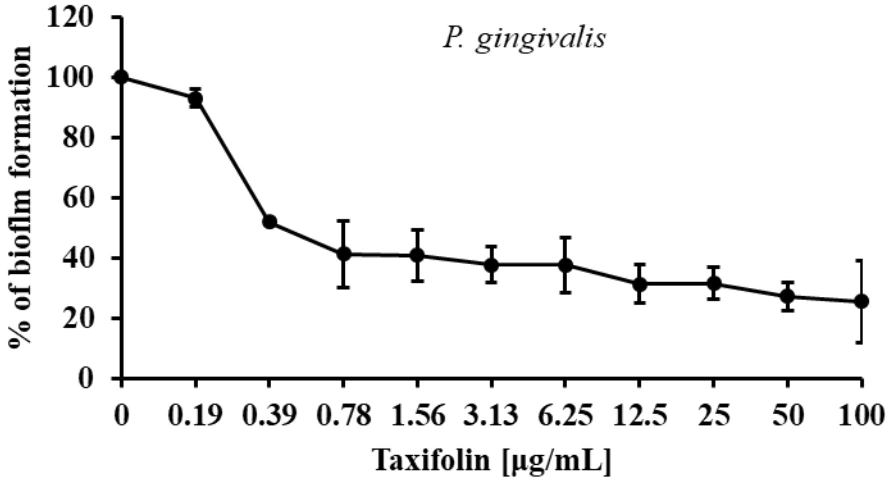
P. gingivalis
120
100
80
60
40
20
0
% of bioflm formation
0
0.19
0.39
0.78
1.56
3.13
6.25
12.5
25
50
100
Taxifolin [µg/mL]

(G)

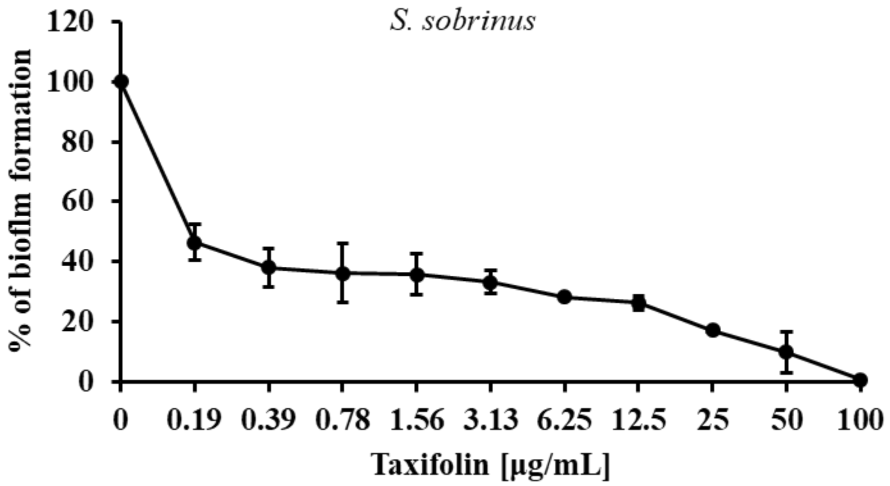
S. sobrinus
% of bioflm formation
120
100
80
60
40
20
0
0
0.19
0.39
0.78
1.56
3.13
6.25
12.5
25
50
100
Taxifolin [μg/mL]
